# If the Doctors Refuse?

**Published:** 1923-10

**Authors:** R. W. Harris


### IF THE DOCTORS REFUSE?

BY R. W. HARRIS.

THE appointment of a new Minister of Health just two or three weeks before the doctors were hoping for an announcement as to the new insurance capitation fee could hardly make for expedition. But Sir William Joynson-Hicks will have had a reasonable opportunity of storing his open mind with the facts relating to the panel doctors' pay. Parliament is in recess. The troublesome housing question is quiescent. There is, so far as can be judged, no immediate health problem which would compete with that of the panel doctors' pay for the first call on the new Minister's time. And he will have the advantage of consultation with the outgoing Minister on the question. How far the doctors will regard it as an advantage that there should be consultation with the new guardian of the public purse may be fairly conjectured without difficulty.

### Legislation Inevitable.

Consultation with the Chancellor of the Exchequer would, doubtless, in any case, be essential if a Bill were contemplated involving any charge on the Exchequer. And it appears inevitable that there should be legislation; unless, indeed, the Minister should propose a 7s. settlement, and adhere to his proposal, notwithstanding the storm. Apart from legislative provisions of a purely temporary character, the amount available for medical benefit is 9s. 6d. This happens to coincide with the present amount of the capitation fee, but is in fact a different figure. It includes provision for drugs and mileage, and leaves about 7s. for the doctors' capitation fee. Legislation, which would become necessary if the settlement were at some higher figure than 7s., would be of the simplest possible character. It would merely provide for the substitution of $x$ shillings for the sum of 9s. 6d. which is charged against insurance funds by the Act of 1920; $x$ shillings would be the total cost of medical benefit including the amount necessary to obtain from the doctors an adequate, reasonably contented service.

### The Chances of Refusal.

The doctors' representatives have put forward a considered statement in support of their view that 10s. 6d. or thereabouts would be an appropriate capitation fee. Will there be an organised withdrawal of service if the Minister insists on some figure less than the present 9s. 6d.? The profession is, undoubtedly, better organised than in 1912. A plan of campaign is in existence. There is a fairly strong defence fund. But it is one thing to get a body of men to refuse to undertake a new business which many of them look upon with suspicion, and quite another thing to persuade them to abandon an assured source of income under a system where the willingness of every client to pay his account is not in question. It may, in any case, be fairly assumed that the Minister will not propose a settlement that will provoke a universal withdrawal of service. It is necessary, however, to see what is the statutory position in the event of a considerable withdrawal.

### The Unpleasant Word, "Strike."

That unpleasant word, "strike," clearly applies to a refusal to carry on the service for the remuneration which Parliament is prepared to sanction. But it would be unfair, in using the word, not to state expressly that it does not connote on the part of the doctors any intention of withholding their services from suffering humanity. The fact that such a course of action has never been contemplated will not prevent the headline writers in certain dailies from writing "Doctors lay down stethoscopes." The doctors' attitude is for them at once a strength and a weakness. It would keep them right with public opinion, but it would also enable the Minister to face a general refusal of his terms with a nearer approach to equanimity.

### The Strike Provisions.

The Minister is not in the position of having been placed, by Parliament, under an obligation to produce an adequate medical service for the insured population under any conditions whatsoever, and regardless of cost. Had that been so he would indeed have been awkwardly situated if the doctors were to stand out for terms which seemed to him unreasonable. His first duty under sec. 15 (2) of the National Insurance Act, 1911, is, it is true, to see that, through the Insurance Committees, arrangements are made under which the insured population receive "adequate medical attendance and treatment"; but this requirement is subject to the words "save as hereinafter provided." And there will be found, at the end of the sub-section, strike provisions which, read in conjunction with section 11 of the Act of 1913 and section 8 of the Act of 1920, will be found to be of a very comprehensive character. I must reserve the examination of these provisions for another article.

### Complete Suspension Unlikely.

In the meantime, it may be pointed out that the Minister's powers extend to a complete suspension of the scheme of medical benefit if such a course appears to him to be necessary. There is hardly likely to be any necessity for such drastic action. It could undoubtedly arise, even if many doctors were prepared to carry on under the panel system, if the withdrawals were so widespread that in no single area were there to be found enough panel doctors to give an adequate service. While, therefore, it is strictly within the Minister's powers to suspend medical benefit for the whole country, or for all save a negligible portion, it may be supposed that public opinion would be averse from the carrying on of the National Health Insurance Scheme without a medical service, and would undoubtedly see in such a situation something which could not be explained away as due to mere tyrannical action on the part of an organised medical profession. What has to be contemplated is the possibility of a breakdown of the panel system in parts, and it will be found on examination that there is adequate provision for meeting such a contingency.

